# Myofibroblastic reaction is a common event in metastatic disease of breast carcinoma: a descriptive study

**DOI:** 10.1186/s13000-014-0196-6

**Published:** 2014-10-22

**Authors:** Xavier Catteau, Philippe Simon, Jean-Christophe Noël

**Affiliations:** Institute of Pathology and Genetics, Gosselies, Belgium; Faculty of Medicine, Université Libre de Bruxelles, Brussels, Belgium; Gynaecology Unit, Erasme University Hospital-Université libre de Bruxelles, Brussels, Belgium; Gynaecopathology Unit, Pathology Department, Erasme University Hospital-Université Libre de Bruxelles, Brussels, Belgium; Department of Pathology, Institute of Pathology and Genetics, 25, Avenue Georges Lemaître, Gosselies, 6041 Belgium

**Keywords:** Myofibroblasts, Breast carcinoma, SMA, CD34, Lymph node, Liver, Metastasis

## Abstract

**Background:**

The modification of stromal components with the disappearance of CD34 positive fibrocytes and by contrast the acquisition of smooth-muscle actin positive myofibroblasts is a frequent event in breast carcinomas but has been little studied in its metastatic sites. Therefore, the aim of the present study is to examine the stromal expression of CD34 and SMA in lymph node and liver metastases which are two of the most frequent metastatic breast cancer sites.

**Methods:**

The distribution of CD34 fibrocytes and SMA myofibroblasts has been studied by immunohistochemistry in 41 lymph node and 36 liver metastases from patients with invasive carcinoma of no special type.

**Results:**

No CD 34 fibrocytes were noted in the stroma of metastasis. By contrast, smooth-muscle actin stromal expression was observed in 95.1% of lymph node and 97.2% of liver metastases, independently of histological features of tumours.

**Conclusions:**

Myofibroblasts represent a major and constant component in the metastatic tumoral stroma of breast carcinoma highlighting that these cells could play an active role in tumour cells proliferation and spread.

**Virtual Slides:**

The virtual slide(s) for this article can be found here: http://www.diagnosticpathology.diagnomx.eu/vs/13000_2014_196

## Background

The importance of the stromal microenvironment has been suggested to play a major role in breast carcinoma by promoting tumour growth, progression and invasion [1-4]. In particular according to these data we and others have clearly demonstrated that the loss of CD34 fibrocytes and acquisition of peritumoral myofibroblasts expressing smooth muscle actin (SMA) is a fundamental step both in ductal carcinoma in situ (DCIS) and invasive carcinoma of no special type (NST) [5,6]. If the acquisition of a myofibroblastic differentiation is an important data in peritumoral connective tissue remodeling [4], the morphological characterization of stromal microenvironment and particularly of myofibroblastic peritumoral cells in metastatic location is less understood. In preliminaries studies, some authors have suggested that the acquisition of a myofibroblastic differentiation could play a role in metastatic colonic adenocarcinoma [7] but however, until now, these data have not been clearly described in breast metastatic sites. Therefore, to clarify this issue, the aim of the present study is to assess by immunohistochemistry, the topographic distribution of CD 34 positive fibrocytes and SMA positive myofibroblasts both in axillary lymph node and liver metastases which are frequent in breast carcinoma and strongly associated with an increased risk of distant metastasis and poor overall survival [8].

## Methods

### Study population

We used a computer database from the Pathology and Genetics Institute (IPG) to identify 77 consecutive patients diagnosed between January 2008 and December 2012 with lymph node (n = 41) and liver metastasis (n = 36). The study protocol was approved by the institutional ethics (Ethics Committee Erasme Hospital) and research review boards. The belgian number (number of agreation) of this committee is OM021. The reference for this study is P2012/191. Consent has been established by the local ethics committee and is in accordance with Belgian and International law. For each patient, the following parameters including age, TNM classification, tumour grade and tumour size were performed according to the 4^th^ edition of WHO classification and are summarized in the Table 1.

**Table 1:** **Clinicopathological data of the study population**

	**Liver metastases N = 36**	**Lymph node metastases N =41**
	No.	No.
Age		
Mean	59.6	59
Range	34 - 86	37 - 86
Primary tumour size		
T1 (0.1- 2 cm)	18	21
T2 (>2- 5 cm)	14	17
T3 (>5 cm)	4	3
Primary tumour grade		
Grade 1	3	8
Grade 2	23	22
Grade 3	10	11

### Immunohistochemistry

The specimens were fixed in histology-grade 4% buffered formalin. Series paraffin sections were stained with haematoxylin and eosin and immunohistochemical detection was performed according to the manufacturer’s protocols (Table 2). We used a fully automated immunohistochemical system (Autostainer Link A48 Dako).

**Table 2:** **Antibodies used in this study**

**Antigen**	**Clone**		**Dilution**	**Source**	**Catalog number**
CD 34	QBEnd-10	Monoclonal Mouse	Ready-to-use	Dako	IR63261
Vimentine	V9	Monoclonal Mouse	Ready-to-use	Dako	IR63061
α-SMA	1A4	Monoclonal Mouse	Ready-to-use	Dako	IR00611
CKAE1/AE3	AE1/AE3	Monoclonal Mouse	Ready-to-use	Dako	IR05361

### Semi-quantitative Assessment of Immunohistochemistry

We compared the distribution of CD34 and SMA between stromal areas located within the metastasis with areas of normal liver and lymph node tissue. The immunoreactivity of CD34 and SMA was assessed semi-quantitatively in the free tissue and the tumour. The percentage of stromal cells expressing each antigen was graded as “0”, “+”, “++”, “+++”, “++++” when up to 5%, more than 5% and up to 25%, more than 25% and up to 50%, more than 50% and up to 75% or more than 75% of stromal cells, disclosed immunoreactivity, respectively. Percentages were assessed by two independent observers, assuming that a high-power microscopic field (objective ×40, microscopic magnification: ×400) harboured 100 stromal cells (range: 75–150) as previously described [9]. The relationship between the staining pattern of SMA and different clinical and histological features (age, tumour size and grade, TNM classification) was compared using a Chi-squared test. A p value <0.05 was considered statistically significant. All analyses were performed using Statistica®.

## Results

### CD 34 and SMA expression in lymph node metastases

**Figure 1:**
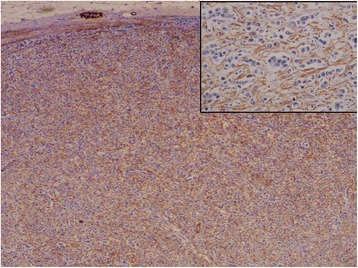
**Typical example of SMA positive myofibroblasts within lymph node metastasis of invasive mammary carcinoma of NST (x100).** Note that the myofibroblasts surround intimately the cancer cells at high power view (inset; x400).

In the normal lymph node, CD34 expression was limited both on the capsule and pericapsular fibrocytes but also in vessels within the parenchyma. CD34 fibrocytes were totally absent in peritumoral stroma and the immunoreactivity was restricted to the vasculature. By contrast, myofibroblasts were present in peritumoral stroma in 95% of cases with in a majority of cases more than 50% of stromal cells positive but this expression was not statistically correlated with clinical or pathological features (p > 0.05) (Tables 3 and 4). The peritumoral myofibroblasts surrounded intimately the malignant cells (Figure 1). In the capsule both in the normal and peritumoral areas, a strong immunoreactivity for SMA was also observed. Lastly, in normal area, the reticular dendritic cells, stromal cells with myoïd features and vascular walls showed as previously described a discrete to moderate reactivity for SMA [10-12].

**Table 3:** **Stromal SMA expression in lymph node and liver metastatic sites**

**SMA expression**	**0**	**+ or ++**	**+++ or ++++**	**Total**
Lymph node	2 (5%)	5 (12%)	34 (83%)	41
Liver	1 (3%)	3 (8%)	32 (89%)	36

**Table 4:** **Relation of SMA stromal expression and clinicopathological features**

	**Lymph node metastases**			**Liver metastases**		
	**Strong expression**	**Weak expression**	**p**	**Strong expression**	**Weak expression**	**p**
Age						
≤ 40	3	0		12	2	
> 40	31	7	0.4	20	2	0.6
Tumour grade						
G1	6	2		2	1	
G2	17	4		21	2	
G3	11	1	0.6	9	1	0.9
Tumour size (mm)						
≤ 10	4	2		3	1	
> 10 and ≤ 20	14	2		12	2	
> 20	16	3	0.4	17	1	0.5

### CD 34 and SMA expression in liver metastases

**Figure 2:**
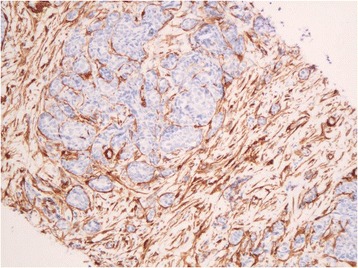
**Myofibroblastic reaction within peritumoral stroma in liver metastasis (x200).**

In normal liver parenchyma, CD 34 expression was limited to vascular walls and focal immunoreactivity in portal tract. In the peritumoral stroma as in the lymph node, the immunoreactivity was restricted to the vasculature. Myofibroblasts are found intimately surrounding tumoral metastatic cells in peritumoral stroma in 97% of cases and like in lymph node was not statistically correlated with clinical and pathological features (Tables 3 and 4) (Figure 2). In normal liver area, as previously described, both hepatic stellate cells (Ito cells) in perisinusoidal spaces, portal tracts and vascular walls were positive for SMA [13].

## Discussion

In preliminary papers, we have previously described that the stromal reaction and in particular tumor-associated myofibroblasts which are prominent in this stroma is a common feature both in situ and invasive breast carcinomas. In the present study, for the first time, we have demonstrated that myofibroblastic reaction was also common both in lymph node and liver metastases. Indeed, in more than 90% of metastatic lymph node and liver metastatic cases, a peritumoral myofibroblastic reaction is present and the myofibroblasts generally surrounded intimately the tumoral cells. In addition, like in primary breast carcinoma, we have not observed CD 34 fibrocytes in the stroma and the immunoreactivity for this marker was restricted to the vascular walls, which possibly represent “neovessels”.

In primary breast carcinoma, we have previously demonstrated that one of the potential origin of this peritumoral myofibroblasts is the transformation of resident fibrocytes CD 34 positive into myofibroblats by the way of the TGF-ß 1/TGF-ß 1 receptor. However, the precursor of these myofibroblasts remains hypothetical in metastatic process.

In liver, we have could demonstrate like others, firstly that myofibroblasts were absent in normal parenchyma and secondly that SMA positivity was observed in vascular walls, portal tract stroma and hepatic perisinusoidal cells [13]. Therefore, as suggested by several authors in liver fibrosis, these cells could be potential precursor for myofibroblasts, which constitute the major source of collagen deposits [14,15]. By analogy, in the lymph node, stromal cells from the capsule, stromal cells with myoïd features and endothelial cells are potential precursors of myofibroblasts. In addition, both in lymph node and liver metastases, generation of myofibroblast either by epithelial-mesenchymal transition (EMT) process from epithelial carcinomatous cells, resident mesenchymal stem cells, or from totipotential bone marrow cells is still debate [16,17].

If the myofibroblastic reaction seems a constant event both in breast carcinoma lymph node and liver metastases, until now, it is unclear whether this event is favourable to development of the metastatic process or by opposition is just a secondary passive reaction remains unsettled. In diverse primary carcinomatous processes including breast carcinoma, myofibroblasts promote tumour growth, invasion and angiogenesis through the paracrine effects of multiple factors including TGF-ß 1 and matrix-metalloproteinases [18]. However, these factors are actually poorly characterized in metastatic process actually.

## Conclusions

In summary, the presence of activated myofibroblasts in lymph node and liver metastases of breast carcinoma highlights the importance of the microenvironment in supporting cancers. Understanding the relationship between myofibroblasts and metastases is not just of prognostic significance, it could provide a new therapeutic target for the treatment of advanced cancer.

## Abbreviations

SMA: Smooth-muscle actin
DCIS: Ductal carcinoma in situ
NST: No special type
TGF-ß: Transforming growth factor-beta
EMT: Epithelial-mesenchymal transition
MMP: Matrix metalloproteinases
